# ACSL1 promotes imatinib-induced chronic myeloid leukemia cell senescence by regulating SIRT1/p53/p21 pathway

**DOI:** 10.1038/s41598-022-21009-6

**Published:** 2022-10-26

**Authors:** Wen Liu, Xiaoying Zhu, Ling Tang, Na Shen, Fanjun Cheng, Ping Zou, Yong You, Guolin Yuan, Qing Li, Xiaojian Zhu

**Affiliations:** 1 Institute of Hematology, Union Hospital, Tongji Medical College, Huazhong University of Science and Technology, Wuhan, 430022 China; 2 Department of Hematology, The First Affiliated Hospital of Zhengzhou University, Zhengzhou, 450000 China; 3 Department of Hematology, Affiliated Hospital of Hubei University of Arts and Science, Xiangyang, 441021 China; 4 Department of Hematology, Wuhan No. 1 Hospital, Wuhan, 430000 China; 5 Department of Hematology, Tongji Hospital, Tongji Medical College, Huazhong University of Science and Technology, Wuhan, 430030 China

**Keywords:** Cancer therapy, Senescence

## Abstract

Although tyrosine kinase inhibitors (TKIs) improve the prognosis of chronic myeloid leukemia (CML) patients, resistance to TKIs and residual leukemia stem cells (LSCs) inevitably become the bottleneck of cure. Therefore, we need to explore novel treatment strategies based on conventional treatment strategies. Our previous study found that CML cell senescence may be one of the main factors to achieve clinical cure of CML. Studies have shown that lipid metabolism plays a key role in cellular senescence. Here, we found that long-chain acyl-CoA synthetase 1 (ACSL1) was significantly up-regulated in senescent CML cells. Furthermore, we demonstrated that overexpression of ACSL1 induces senescence and inhibits cell growth in K562 cells by altering cell cycle progression, and enhances the proliferation-inhibiting effect of imatinib. Overexpression of ACSL1 enhances imatinib-induced tumorigenic decline in K562 cells in vivo. Knockdown of ACSL1 reverses imatinib-induced senescence in K562 cells. Mechanistically, overexpression of ACSL1 induced senescence in K562 cells via the SIRT1/p53/p21 axis. Collectively, our study showed that ACSL1 promotes imatinib-induced K562 cells senescence and tumor growth by regulating SIRT1/p53/p21 pathway. The ACSL1/SIRT1/p53 signal axis is a novel mechanism of cell senescence in CML and a new potential target for eradication of CML LSCs.

## Introduction

Chronic myeloid leukemia (CML) is caused by the acquisition of the fusion gene BCR-ABL1 in hemopoietic stem cells, which is transformed into leukemia stem cells (LSCs) and is characterized by Philadelphia (Ph) chromosome rearrangements and the constitutive expression of the fusion protein BCR–ABL1^1^. The advent of tyrosine kinase inhibitors (TKIs) targeting the kinase activity of BCR–ABL1 has transformed CML from a fatal disease to a controllable one for the vast majority of patients^2^. However, due to TKI resistance and the persistence of TKI-insensitive quiescent leukemia stem cells (LSCs), some patients fail to achieve a complete cytogenetic response (CCR) or relapse after remission. Therefore, we need to explore novel treatment strategies based on conventional treatment strategies.

Cellular senescence is a state of irreversible growth arrest that results in a senescent phenotype, which is characterized by a typical flat and enlarged shape, upregulated senescence-associated β-galactosidase (SA-β-gal) activity, formation of senescence-associated heterochromatin foci (SAHF), cell cycle arrest, and inhibited cell proliferation: senescence induction may be an effective approach to treat cancer^3,4^. Our previous study showed that the FOLR3 SNP rs139130389 may be involved in treatment free remission (TFR) by activating mitochondria to drive CML LSCs proliferation and replication-induced cellular senescence^5^.

Lipids have long been recognized for their role as the basic components of cells and form a permeable barrier that defines cells and internal compartments. An emerging body of data suggest that lipids play an important role in central signaling and structural molecules for various cell fates, such as cellular senescence^6^. The long-chain acyl-CoA synthase (ACSL) family is involved in the regulation of fatty acids. ACSLs can convert fatty acids into acyl-CoA, which can be oxidized to generate energy, and can be incorporated into triacylglycerols (TAG) and phospholipids for storage and membrane biogenesis, respectively^7^. The different ACSLs are related to the distribution and oxidation of intracellular fatty acids in the storage pathway. The long-chain acyl-coenzyme A synthetases 1 (ACSL1) is a member of the ACSL family. However, no study has explored whether ACSL1 induces senescence in CML cells. Therefore, we hypothesized that ACSL1 may induce the senescence of CML cells.

Either or both of the p53/p21 and p16/Rb tumor suppressive pathways, respond to stimulations that induce cellular senescence establish and/or maintain the senescence growth arrest^8,9^. There are multiple upstream regulators, downstream effectors and modified side branches in both pathways, they also regulate several other features of senescent cells, such as SASP and cell proliferation. However, K562 cells is known to be p53-null type^10–12^. Therefore, we constructed K562 cell line expressing the wild-type p53 gene for further mechanistic studies.

In this study, we reported for the first time that a higher ACSL1 level exists in senescent K562 cells and increased ACSL1 promotes imatinib-induced chronic myeloid leukemia cell senescence by regulating SIRT1/p53/p21 pathway.

## Results

### ACSL1 is up-regulated in senescent K562 cells

**Figure 1:**
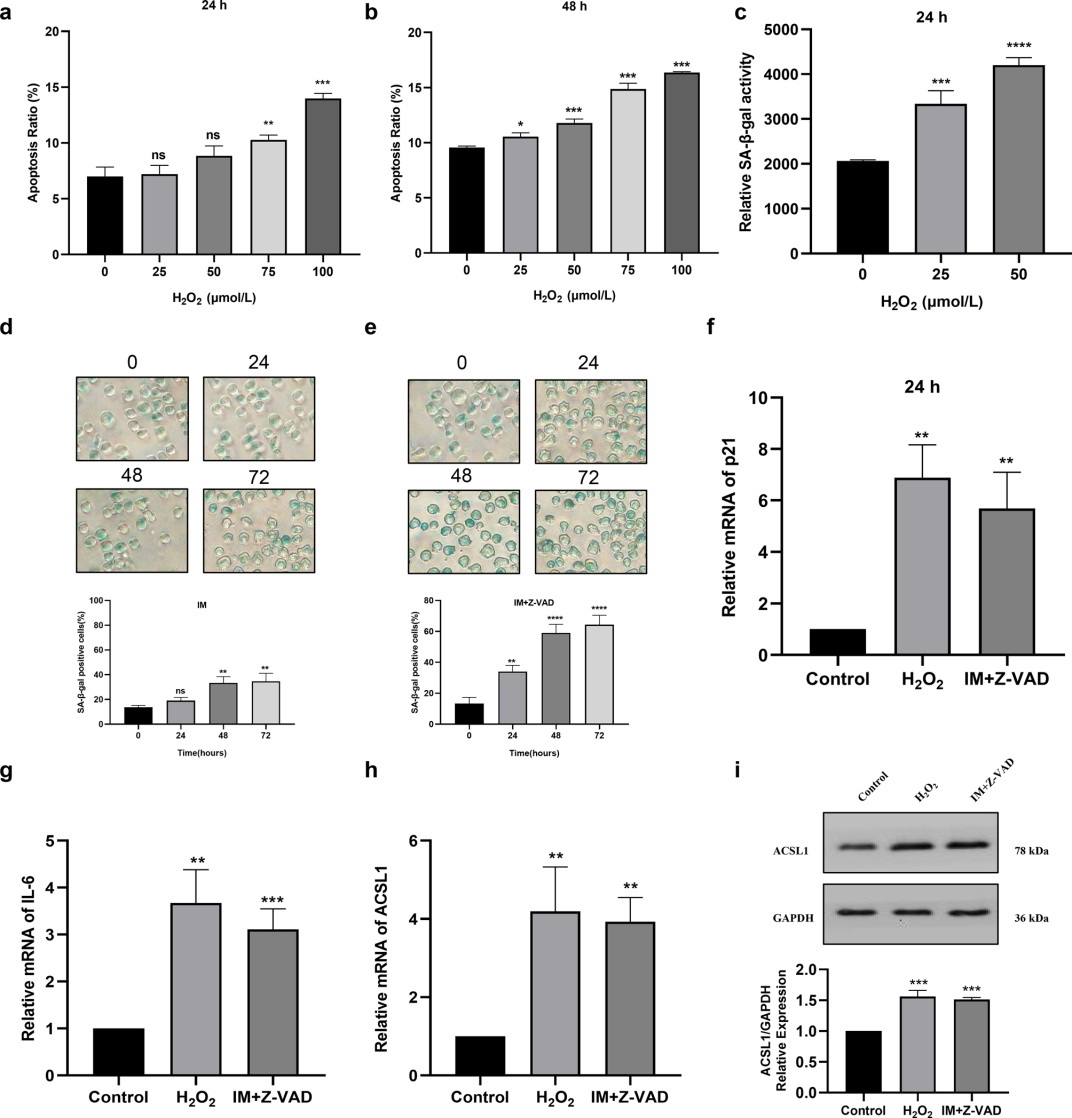
ACSL1 is up-regulated in senescent K562 cells. (**a**–**c**) Apoptosis and the mean fluorescence intensity (MFI) of SPiDER-β Gal were measured by flow cytometry to determine the optimal concentration and treatment time of H_2_O_2_ in K562 cell line. (**d**,**e**) Cell senescence assay by SA-β-gal staining was applied to determine the optimal treatment time of imatinib plus Z-VAD in K562 cell line. (**f**,**g**) qRT-PCR was applied to quantify the relative expression of senescence-associated genes p21 and IL-6. (**h**,**i**) qRT-PCR and western blot analysis were applied to quantify the relative mRNA and protein expression of ACSL1. *ns* not significant, *p < 0.05, **p < 0.01 and ****p < 0.0001.

First, we constructed K562 cell line expressing the wild-type p53 gene (Figure S1). Then, K562 cell senescence models induced by H_2_O_2_ and imatinib were used to explore the role of ACSL1 in CML cellular senescence. We determined the optimal concentration and treatment time of H_2_O_2_ in the establishment of K562 cell senescence model by apoptosis experiments. We observed that H_2_O_2_ did not cause significant apoptosis at 25 μmol/L and 50 μmol/L for 24 h compared with other groups (Fig. 1a,b). Then, we found that H_2_O_2_ can further promote K562 cell senescence at 50 μmol/L for 24 h than at 25 μmol/L (Fig. 1c). Therefore, 50 μmol/L of H_2_O_2_ was chosen for further study. In addition, studies have shown that inhibition of imatinib-induced apoptosis in K562 cells can increase the SA-β-gal-positive senescent cell population^13^. Therefore, we treated K562 cells with 1 μmol/L imatinib or combined with the pan-caspase inhibitor Z-VAD-FMK (Z-VAD). The results showed that compared with other groups, imatinib plus Z-VAD (30 min pretreatment) incubated with K562 for 48 h could significantly induce K562 cells senescence (Fig. 1d,e). Then we detected the expression of senescence-related genes p21 and IL-6 mRNA by qRT-PCR. The results showed that the expression of p21 (Fig. 1f) and IL-6 (Fig. 1g) was increased in K562 cells treated with H_2_O_2_ or imatinib plus Z-VAD compared to the control group. These results further indicated that the cellular senescence models were successfully constructed. We examined the expression levels of ACSL1 mRNA and protein in two cellular senescence models by qRT-PCR. The results showed that the expression levels of ACSL1 mRNA (Fig. 1h) and protein (Fig. 1i) were significantly increased in two K562 cell senescence models compared with the control group. These results revealed that high expression of ACSL1 is related to the cellular senescence in CML.

### Overexpression of ACSL1 mediates imatinib-induced senescence in K562 cells

**Figure 2:**
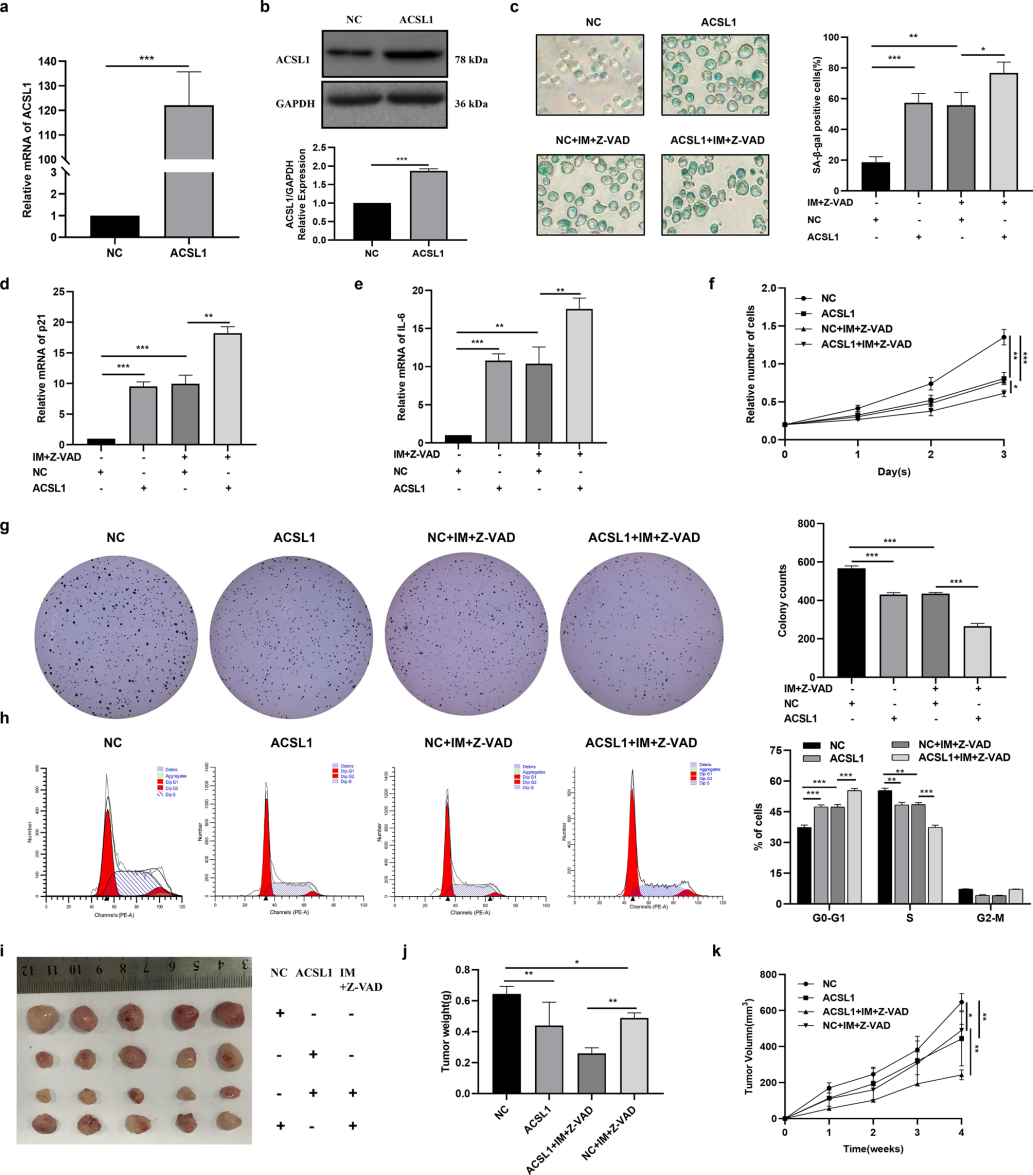
Overexpression of ACSL1 mediates imatinib-induced senescence in K562 cells. (**a**,**b**) qRT-PCR and western blot analysis verified efficiency of overexpressing ACSL1. (**c**) Cell senescence analysis determined by SA-β-gal staining. (**d**,**e**) qRT-PCR was applied to quantify the relative expression of senescence-associated genes p21 and IL-6. (**f**) Cell proliferation analysis determined by CCK8 assay. (**g**) Cloning formation assay indicating the in vitro growth of K562 cells. (**h**) Cell cycle distribution analyzed using PI staining followed by flow cytometry. (**i**) Representative images of tumor from a Xenograft model in nude mice. (**j**) Tumor weight of all mice in each group. (**k**) Tumor volumes were measured on the indicated days. The data are presented as the mean ± SD of three independent experiments. *p < 0.05, **p < 0.01 and ***p < 0.001.

To further investigate the relationship between ACSL1 and imatinib in K562 cells senescence, we constructed K562 cells overexpressing ACSL1 by lentiviral transfection (Fig. 2a,b). Then, we treated NC K562 cells and K562 cells overexpressing ACSL1 with IM plus Z-VAD, respectively. Cellular senescence is characterized by upregulated SA-β-gal activity and senescence-related gene expression, inhibited cell proliferation, cell cycle arrest and formation of SAHF. We performed SA-β-Gal staining to detect intracellular SA-β-Gal activity. The results showed that compared with the control group, overexpression of ACSL1 or IM plus Z-VAD could increase the proportion of SA-β-Gal positive cells (Fig. 2c). Then we detected the mRNA expression of two typical senescence-related genes, IL-6 and p21, by qRT-PCR. The results showed that compared with the control group, overexpression of ACSL1 or IM plus Z-VAD could up-regulate the mRNA expressions of senescence-related genes p21 (Fig. 2d) and IL-6 (Fig. 2e). CCK8 and clonogenic assays were performed to evaluate the cell proliferation and clonogenic capacity, respectively. The results showed that overexpression of ACSL1 or IM plus Z-VAD could inhibit the proliferation of K562 cells (Fig. 2f) and decrease the ability of colony formation (Fig. 2g) compared with the control group. Cell cycle was assessed with flow cytometry and the results revealed that compared with the control group, overexpression of ACSL1 or IM plus Z-VAD induced K562 cell cycle arrest (Fig. 2h). Compared with the IM plus Z-VAD group, overexpression of ACSL1 could further promote the above effects of IM plus Z-VAD on K562 cells. All these characteristics accord with classical cellular senescence markers. Taken together, these results revealed that overexpression of ACSL1 mediates imatinib-induced senescence in K562 cells. In addition, we also found that overexpression of ACSL1 mediates imatinib-induced decrease in tumorigenicity of K562 cells in vivo (Fig. 2i–k)*.*

### Knockdown of ACSL1 reverses imatinib-induced K562 cells senescence

**Figure 3:**
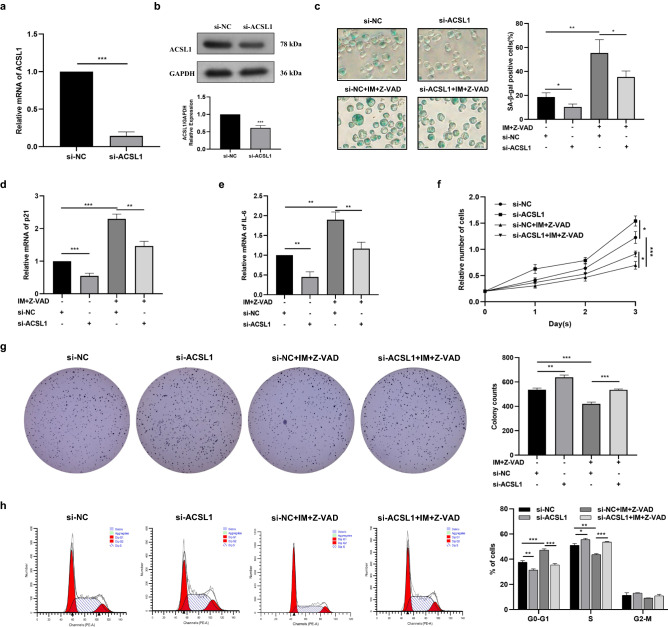
Knockdown of ACSL1 expression reverses imatinib-induced senescence in K562 cells. (**a**,**b**) Cells were transfected with si-NC or si-ACSL1. (**c**) Cell senescence analysis determined by SA-β-gal staining. (**d**,**e**) qRT-PCR was applied to quantify the relative expression of senescence-associated genes p21 and IL-6. (**f**) Cell proliferation analysis determined by CCK8 assay. (**g**) Cloning formation assay indicating the in vitro growth of K562 cells. (**h**) Cell cycle distribution analyzed using PI staining followed by flow cytometry. The data are presented as the mean ± SD of three independent experiments. *p < 0.05, **p < 0.01 and ***p < 0.001.

We transfected K562 cells with small interfering RNA (siRNA) targeting ACSL1 to knock down the expression of ACSL1 under the IM plus Z-VAD conditions, siRNA-3 had the best knockdown efficiency and was used for subsequent experiments (Fig. 3a,b). We assessed intracellular SA-β-Gal activity, mRNA expression of IL-6 and p21, cell proliferation, clonogenic capacity, cell cycle. The results showed that compared with the control group, knockdown of ACSL1 reduced the proportion of senescent K562 cells (Fig. 3c), down-regulated the expression of p21 (Fig. 3d) and IL-6 (Fig. 3e) mRNA, enhanced the proliferation ability (Fig. 3f), improved the ability of colony formation (Fig. 3g), and increased the proportion of cells in S phase (Fig. 3h). Correspondingly, IM plus Z-VAD had opposite effects on K562 cells. Compared with the imatinib plus Z-VAD group, knockdown of ACSL1 could partially reverse the above effects of IM plus Z-VAD on K562 cells.

### ACSL1 induces senescence through SIRT1/p53/p21 pathways in K562 cells

**Figure 4:**
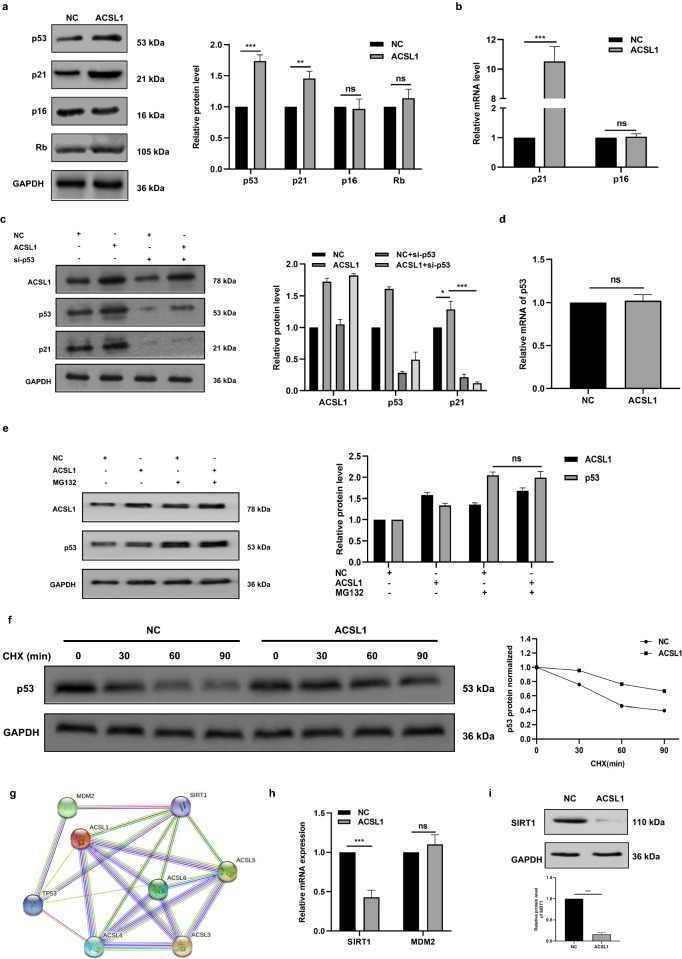
ACSL1 induces senescence through SIRT1/p53/p21 pathways in K562 cells. (**a**) Western blot analysis of the expression levels of senescence related proteins (p53, p21, p16, Rb) after transfection with ACSL1 in K562 cells. (**b**) qRT-PCR was applied to quantify the relative mRNA expression of p21 and p16. (**c**) Western blot detected the expression levels of ACSL1, p53, and p21 in four groups K562 cells. (**d**) qRT-PCR was applied to quantify the relative mRNA expression of p53. (**e**) Western blot assays for ACSL1 and p53 expression in K562 NC and ACSL1 cells treated with MG132. (**f**) Western blot assays for p53 expression in K562 NC and ACSL1 cells treated with CHX. (**g**) The protein–protein networks view from STRING database showing the networks of ACSL1, p53, and SIRT1. (**h**) The mRNA levels of SIRT1 and MDM2 after transfection with ACSL1 in K562 cells. (**i**) The protein levels of SIRT1 after transfection with ACSL1 in K562 cells. *p < 0.05, **p < 0.01 and ***p < 0.001.

The p53/p21 and p16/Rb signaling axes are considered to be the most important signaling pathways in tumor cell senescence. Therefore, we first detected p53, p21, p16 and Rb protein expression in K562 cells overexpressing ACSL1 by Western-Blot assay. Compared with control group, overexpressed ACSL1 induced the upregulation of p53 and p21 protein but did not affect the protein expression of p16 and Rb (Fig. 4a). qRT-PCR results also showed that p21 mRNA expression was increased while p16 mRNA expression was unchanged in cells overexpressing ACSL1 (Fig. 4b). p53 is considered to be the main transcription factor regulating p21 expression. To confirm that ACSL1 regulates p21 through p53, we transfected si-p53 in K562 cells overexpressing ACSL1 and in NC (K562) as control. The results showed that overexpression of ACSL1 could not increase the expression of p21 after knockdown of p53 (Fig. 4c). These results suggested that overexpression of ACSL1 promotes K562 cells senescence by activating the p53/p21 pathway.

Studies have shown that ACSL1 mainly regulates downstream molecules at the transcriptional level^14^. To further investigate the mechanism by which ACSL1 regulates p53, we examined p53 mRNA expression in K562 cells overexpressing ACSL1. The results showed that the levels of p53 mRNA was not up-regulated after overexpression of ACSL1 (Fig. 4d). We further examined the post-transcriptional regulation of p53 in K562 cells overexpressing ACSL1. We treated K562 cells overexpressing ACSL1 with the proteasome inhibitor MG132 or the protein synthesis inhibitor cycloheximide (CHX). The results showed that overexpression of ACSL1 did not increase the p53 protein expression in cells treated with MG132 (Fig. 4e). Overexpression of ACSL1 resulted in an increase in the half-life of p53 protein compared to the control group (Fig. 4f). These data suggested that overexpression of ACSL1 upregulates p53 at the post-translational level by increasing p53 protein stabilization, prolonging protein half-life.

SIRT1 and MDM2 are the main upstream regulatory proteins of p53. Therefore, we first performed the analysis through the STRING database. The results showed that it was SIRT1 that had a direct interaction with ACSL1 (Fig. 4g). SIRT1, a NAD^+^-dependent type III histone/protein deacetylase, plays a key role in cellular senescence. SIRT1 is involved in the regulation of p53 function via deacetylation. The results of qRT-PCR (Fig. 4h) and Western blot (Fig. 4i) assays also indicated that overexpression of ACSL1 could reduce the expression of SIRT1. These results revealed that ACSL1 induces senescence through SIRT1/p53/p21 pathways in K562 cells.

## Discussion

There is increasing evidence that leukemia stem cells (LSCs) and other cancer stem cells can be induced to senescence. Retinoic acid and/or arsenic trioxide cure patients with acute promyelocytic leukemia (APL) through eradicating APL-initiating cells by activating the p53 and promyelocytic leukemia proteins to induce senescence but not apoptosis of leukemia-initiating cells^15^. In acute myeloid leukemia (AML), the expression of miR-34c-5p promotes eradication of AML LSCs through induced LSCs senescence via p53-p21Cip1-CDK/Cyclin or p53-independent CDK/Cyclin pathways^16^. The senescence induction programs may provide a treatment strategy to eradicate LSCs and inhibit leukemia recurrence and resistance.

Our previous research for the first time found that compared with CD34+ cells from cord blood, healthy mobilization and non-treated CML patients, CD34+ cells in CML patients treated with TKI presented more senescence phenotypes. In addition, the longer TKI treatment lasted, the higher of β-gal activity exhibited, and the fewer colonies produced^5^. The results of colony-forming assay in vitro and tumor xenografts experiment in vivo in this study further confirmed that the tumor formation ability of aging cancer cells has decreased.

An emerging body of data suggested that lipid metabolism is associated with the aging process. The epsilon 2 and epsilon 4 alleles of apolipoprotein E are associated with extreme longevity and late-onset neurodegenerative disease, respectively^17^. In humans, blood triglyceride levels tend to increase, while blood lysophosphatidylcholine levels tend to decrease with age^18^. It has been reported that long-chain acyl-CoA synthetases (ACSL) family displays an essential role in lipid synthesis^19^. ACSLs are key enzymes, they participated in the process of long-chain fatty acids transforming into their acyl-CoA derivatives. However, as a member of the ACSL family, the relationship between ACSL1 and aging is still unknown. Here, we found that a higher ACSL1 level exists in senescent K562 cells and increased ACSL1 promotes imatinib-induced chronic myeloid leukemia cell senescence.

Among the currently known cellular senescence regulatory networks, p53/p21 and p16/Rb are the most important signal transduction pathways. It has been discovered that a variety of oncogenes and tumor suppressor genes play a regulatory role on cellular senescence through the above two signal pathways^20^. In our study, we found that increased ACSL1 expression led to a significant up-regulation of p53 and p21, suggesting that ACSL1 is involved in the regulation of p53/p21 signaling pathway. On the other hand, we found that ACSL1 has no significant effect on the p16/Rb signaling pathway in CML cells. Therefore, we initially confirmed that ACSL1 mainly regulates CML cell senescence through the p53/p21 signaling pathway.

SIRT1 is a mammalian NAD+-dependent deacetylase that exerts multiple effects on cellular metabolism, DNA repair, and senescence by deacetylating a variety of un-histone proteins, including p53^21^. As a classical regulatory protein upstream of p53, SIRT1 promotes the degradation of p53 protein by binding to p53 at the post-transcriptional level, thereby regulating p53 and its downstream signaling pathways. Therefore, we hypothesized that ACSL1 regulates the p53/p21 signaling pathway by targeting SIRT1, thereby affecting the senescence, cell cycle and proliferation of CML cells. Our results that overexpression of ACSL1 decreased the expression of SIRT1 protein supported the hypothesis. In summary, we reported that the increased ACSL1 expression promotes senescence by regulating the SIRT1/p53/p21 signal axis to inhibit tumor proliferation in CML.

In conclusion, our study first elucidated that ACSL1 promotes imatinib-induced K562 cells senescence and tumor growth by regulating SIRT1/p53/p21 pathway. The ACSL1/SIRT1/p53 signal axis is a novel mechanism of cell senescence in CML and may a new potential target for eradication of CML LSCs.

## Methods

### Cell culture

A human CML cell line, K562, was obtained from China Center for Type Culture Collection (Wuhan, China) and cultured in the RPMI-1640 medium (Gibco, USA) supplemented with 10% fetal bovine serum (Gibco, Australia origin) and 1% penicillin/streptomycin (Thermo Fisher Scientific) in a humidified incubator at 37 °C with 5% CO_2_.

### Apoptosis detection

K562 cells (1 × 10^5^) were stained with Annexin-V-FITC/PI (BD Pharmingen) for 15 min at room temperature. All analyses were carried out on a flow cytometer (BD LSRFortessaTM X-20). The results were analyzed on FlowJo V10 (Stanford University, San Francisco, CA, USA).

### Analysis of SA-β gal activity via flow cytometry

SA-β-gal activity was detected using a Cellular Senescence Detection Kit-SPiDER-β Gal (Dojindo, Shanghai, China). K562 cells (1 × 10^5^) were treated with Bafilomycin A1 for 2 h at 37 °C, 5% CO_2_, then stained with SPiDER-β Gal for 30 min at the same conditions. All analyses were carried out on a flow cytometry (BD LSRFortessaTM X-20). The results were analyzed on FlowJo V10 (Stanford University, San Francisco, CA, USA).

### SA-β-gal staining assays

SA-β-gal activity was measured following the manufacturer’s instructions (Beyotime Biotechnology Ltd, Shanghai, China). K562 cells were first fixed with β-galactosidase staining fixative for 15 min at room temperature. Then add staining working solution, incubate at 37 °C overnight, and observe under light microscope.

### RNA extraction and quantitative real-time PCR (qRT-PCR)

Total RNA was extracted from cells using Trizol reagent (Takara, Dalian, China). cDNA was synthesized by reverse transcription using a PrimeScript RT Reagent Kit (Takara, Dalian, China) according to the manufacturer's instructions. qRT-PCR was carried out using the SYBR Premix Ex TaqTM kit (Takara, Dalian, China) and performed on a StepOne Plus real-time PCR system (Life Technologies, Carlsbad, CA). The control gene was GAPDH. The fold changes in mRNA were calculated through relative quantification (2^-ΔΔCt^). The primer sequences were showed in Table S1.

### Cell cycle analysis

K562 cells (1 × 10^6^) were fixed with 70% cold absolute ethyl alcohol overnight. Then, the cells were stained with a mixture of PI and RNAase (BD Pharmingen, USA) at room temperature in the dark for 15 min and analyzed via flow cytometry (BD LSRFortessaTM X-20). The results were analyzed using ModFit LT software (Verity Software House, ME, USA).

### Colony-forming assay

The 6-well culture plates were pre-coated with 2 mL of 1.2% agarose gel (BBI Life Sciences, Shanghai, China) mixed with cell culture medium (bottom layer). Then K562 cells (0.3 × 10^4^) were suspended in 1 mL 0.7% agarose gel mixed with cell culture medium and added onto the bottom layer. Surrounding wells were supplemented with 1 mL PBS. Put the 6-well plates in the incubator after the gel has solidified. Clones were stained with MTT and scored after 10–14 days of incubation at 37 °C and 5% CO_2_.

### Cell counting kit-8 (CCK-8) assay

Cell proliferation was quantified with the CCK-8 assay according to the following protocols. Approximately 5 × 10^3^ cells were plated per well into a 96-well plate and incubated at 37 °C. After incubation with 10 μL of CCK-8 reagent (DOJINDO Laboratories, Kumamoto, Japan) for 2 h at 37 °C in the dark, the optical density was measured at 450 nm using a microtiter plate reader.

### Cell transfection

K562 cells (2 × 10^5^) were pre-seeded onto plates coated with RetroNectin (Takara, Japan) overnight. Lentiviral vectors (Genechem, Shanghai, China) were added to the cell suspension and incubated for 8 h before changing the medium. qRT-PCR and/or Western blotting were performed to verify the relative genes expression. K562 cells transfected with wild-type p53 or ACSL1 were selected for 1–2 weeks with 2 mg/mL neomycin or puromycin, respectively. siRNAs were synthesized and purified by GenePharma (Shanghai, China) and transfected with Lipofectamine 3000 (Invitrogen, Carlsbad, CA, USA) following the manufacturer’s guidelines. The sequence of siRNAs are as follows: si-ACSL1:5′-GCT GAT TGA CAT TCG GCA GTA-3′; si-p53:5′-GAC UCC AGU GGU AAU CUA CTT-3′.

### Western blotting analysis

The proteins were extracted in RIPA lysis buffer (Thermo Scientific) and determined using BCA Protein assay kit (Beyotime, China). Cell lysates were separated on SDS-PAGE gels by electrophoresis and transferred onto polyvinylidene difluoride (PVDF) membranes (Millipore, Eschborn, Germany). The membranes were blocked in 5% milk, and were then washed twice with TBST for 45 min. The total proteins were incubated with primary antibodies at 4 °C overnight and were then washed twice with TBST for 45 min. The membranes were blocked for 1 h in the specific HRP-conjugated secondary antibodies at room temperature. All images were obtained by using BioSpectrum 600 Imaging System (UVP, CA, USA). Antibodies against ACSL1 (Abcam, Cat. No. ab177958), SIRT1 (Abcam, Cat. No. ab183660), p53 (Abcam, Cat. No. ab32389), p21 (Abcam, Cat. No. ab109520), p16^INK4A^ (Abcam, Cat. No. ab201980), Rb (Abcam, Cat. No. ab181616), GAPDH (Proteintech, Cat No. 60004-1-Ig), HRP-conjugated secondary goat anti-mouse (Proteintech, Cat. No: SA00001-1) and goat anti-rabbit (Proteintech, Cat. No: SA00001-2).

### Tumor xenografts

We chose 4-week-old BALB/c nude mice for tumor xenografts experiments. K562 cells were subcutaneously injected into the upper back of the nude mice (1 × 10^7^, 200 μL). Tumor volumes were measured using an external caliper and calculated using the equation: (L × W^2^)/2. Mice were sacrificed and detected for tumor weight after 1 month.

### Statistical analysis

All data are presented as the mean ± SD. Statistical analyses were performed using GraphPad Prism 8.01 software (GraphPad Prism Inc., La Jolla, USA) and SPSS 19.0 statistical software (SPSS). For variables with a normal distribution, unpaired Student’s t test was used to determine the significance of differences between two groups, whereas one-way ANOVA was used for comparisons among three or more groups. p < 0.05 was accepted as statistically significant.

### Ethical approval

All procedures were approved by the Animal Care Committee of Tongji Medical College and all experiments were performed in accordance with relevant guidelines and regulations, in compliance with the ARRIVE guidelines (http://www.nc3rs.org.uk/page.asp?id=1357).

## Supplementary Information

Supplementary Information.

## Data Availability

The datasets used and/or analysed during the current study available from the corresponding author on reasonable request.

## References

[1] Hochhaus A, Baccarani M, Silver RT (2020). European LeukemiaNet 2020 recommendations for treating chronic myeloid leukemia. Leukemia.

[2] Hochhaus A, Larson RA, Guilhot F (2017). Long-term outcomes of imatinib treatment for chronic myeloid leukemia. N. Engl. J. Med..

[3] Omer A, Barrera MC, Moran JL (2020). G3BP1 controls the senescence-associated secretome and its impact on cancer progression. Nat. Commun..

[4] Herranz N, Gil J (2018). Mechanisms and functions of cellular senescence. J. Clin. Invest..

[5] Shen N, Liu T, Liu W (2021). A folate receptor 3 SNP promotes mitochondria-induced clonogenicity of CML leukemia cells: Implications for treatment free remission. Clin. Transl. Med..

[6] Lizardo DY, Lin YL, Gokcumen O (2017). Regulation of lipids is central to replicative senescence. Mol. Biosyst..

[7] Coleman RA, Lewin TM, Muoio DM (2000). Physiological and nutritional regulation of enzymes of triacylglycerol synthesis. Annu. Rev. Nutr..

[8] Ampisi J, D’Adda DFF (2007). Cellular senescence: When bad things happen to good cells. Nat. Rev. Mol. Cell Biol..

[9] Adams PD (2009). Healing and hurting: Molecular mechanisms, functions, and pathologies of cellular senescence. Mol. Cell..

[10] Usuda J, Inomata M, Fukumoto H (2003). Restoration of p53 gene function in 12-O-tetradecanoylphorbor 13-acetate-resistant human leukemia K562/TPA cells. Int. J. Oncol..

[11] Bi S, Hughes T, Bungey J (1992). p53 in chronic myeloid leukemia cell lines. Leukemia.

[12] Boettcher S, Miller PG, Sharma R (2019). A dominant-negative effect drives selection of TP53 missense mutations in myeloid malignancies. Science.

[13] Drullion C, Trégoat C, Lagarde V (2012). Apoptosis and autophagy have opposite roles on imatinib-induced K562 leukemia cell senescence. Cell Death Dis..

[14] Zhang Q, Xie T, Mo G (2021). ACSL1 inhibits ALV-J replication by IFN-signaling and PI3K/Akt pathway. Front. Immunol..

[15] Ablain J, Rice K, Soilihi H (2014). Activation of a promyelocytic leukemia-tumor protein 53 axis underlies acute promyelocytic leukemia cure. Nat. Med..

[16] Peng D, Wang H, Li L (2018). miR-34c-5p promotes eradication of acute myeloid leukemia stem cells by inducing senescence through selective RAB27B targeting to inhibit exosome shedding. Leukemia.

[17] Schachter F, Faure-Delanef L, Guenot F (1994). Genetic associations with human longevity at the APOE and ACE loci. Nat. Genet..

[18] Mapstone M, Cheema AK, Fiandaca MS (2014). Plasma phospholipids identify antecedent memory impairment in older adults. Nat. Med..

[19] Teodoro BG, Sampaio IH, Bomfim LH (2017). Long-chain acyl-CoA synthetase 6 regulates lipid synthesis and mitochondrial oxidative capacity in human and rat skeletal muscle. J. Physiol..

[20] Chen L, Yang R, Qiao W (2019). 1,25-Dihydroxyvitamin D exerts an antiaging role by activation of Nrf2-antioxidant signaling and inactivation of p16/p53-senescence signaling. Aging Cell.

[21] Ong A, Ramasamy TS (2018). Role of Sirtuin1-p53 regulatory axis in aging, cancer and cellular reprogramming. Ageing Res. Rev..

