# Supplementary material for: ACSL1 promotes imatinib-induced chronic myeloid leukemia cell senescence by regulating SIRT1/p53/p21 pathway
Source: Sci Rep. 2022 Oct 26;12:17990. doi: 10.1038/s41598-022-21009-6 (PMC9606008; doi:10.1038/s41598-022-21009-6)
Supplement: Supplementary file 1 — Supplementary Information. [file 41598_2022_21009_MOESM1_ESM.pdf]

ACSL1 promotes imatinib-induced chronic myeloid leukemia cell senescence by regulating SIRT1/p53/p21 pathway

Wen Liu[1], Xiaoying Zhu[1], Ling Tang[1], Na Shen[2], Fanjun Cheng[1*], Ping Zou[1], Yong You[1], Guolin Yuan[3*], Qing Li[4*], Xiaojian Zhu[5]

[1]Institute of Hematology, Union Hospital, Tongji Medical College, Huazhong University of Science and Technology, Wuhan 430022, China
[2]Department of Hematology, The First Affiliated Hospital of Zhengzhou University, Zhengzhou 450000, China
[3]Department of Hematology, Affiliated Hospital of Hubei University of Arts and Science, Xiangyang, China
[4]Department of Hematology, Wuhan No.1 Hospital, Wuhan 430000, China
[5]Department of Hematology, Tongji Hospital, Tongji Medical College, Huazhong University of Science and Technology, Wuhan 430030, China

Corresponding authors:

Qing Li, Department of Hematology, Wuhan No.1 Hospital, Wuhan 430000, China. E-mail: liqing54070907@163.com; Telephone: +86 13627100960.
Guolin Yuan, Affiliated Hospital of Hubei University of Arts and Science, Department of Hematology, Xiangyang Hubei, China. E-mail: yuanguo1973@163.com.
Fanjun Cheng, Institute of Hematology, Union Hospital, Tongji Medical College, Huazhong University of Science and Technology, No.1277 Jiefang Road, Wuhan 430022, China. Email: chengfanjun001@sina.com. Telephone: +86 13807186956.

Figure S1

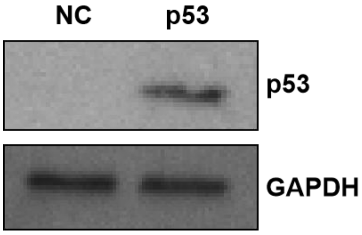

Fig. S1. p53 expression K562 cells. Western blot analysis of p53 in K562 cells.

Table S1. Primer sequences.

Amplicon	Primer FW (5′–3′)	Primer RV (5′–3′)
ACSL1	CTTATGGGCTTCGGAGCTTTT	CAAGTAGTGCGGATCTTCGTG
p16	TTCGCTAAGTGCTCGGAGTTAATAG	ACCCTGTCCCTCAAATCCTCTG
p21	CACTCCAAACGCCGGCTGATCTTC	TGTAGAGCGGGCCTTTGAGGCCCTC
p53	CTTTGAGGTGCGTGTTTGTGC	GGTTTCTTCTTTGGCTGGGGA
SIRT1	GAGTGGCAAAGGAGCAGA	TCTGGCATGTCCCACTATC
MDM2	GAATCATCGGACTCAGGTACATC	TCTGTCTCACTAATTGCTCTCCT
GAPDH	GGGAGCCAAAAGGGTCATCA	TGATGGCATGGACTGTGGTC

Figure S2

Figure 1i

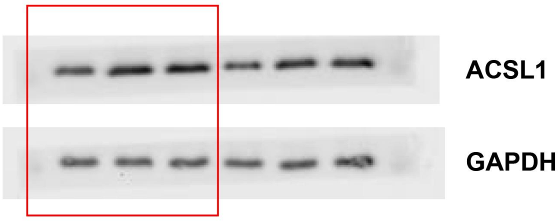

Supplementary Figure S2.

Gels and blots which are shown cropped in Figure 1i. Note: The blot was cut into multiple strips following the protein transfer. The edges of the PVDF membrane are visible. Here, bands containing the 60-90 kDa and 20-40 kDa regions were imaged, respectively.

Figure S3

Figure 2b

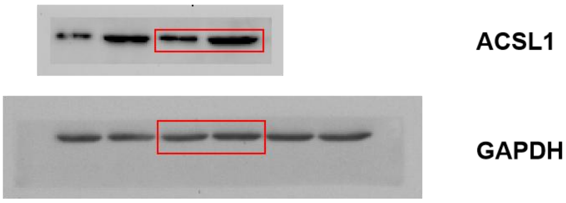

Supplementary Figure S3.

Gels and blots which are shown cropped in Figure 2b. Note: The blot was cut into multiple strips following the protein transfer. The edges of the PVDF membrane are visible. Here, bands containing the 60-90 kDa and 20-40 kDa regions were imaged, respectively.

Figure S4

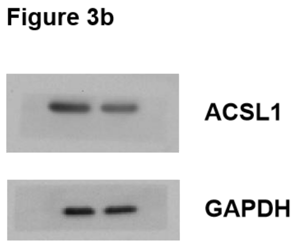

Supplementary Figure S4.

Gels and blots which are shown cropped in Figure 3b. Note: The blot was cut into multiple strips following the protein transfer. The edges of the PVDF membrane are visible. Here, bands containing the 60-90 kDa and 20-40 kDa regions were imaged, respectively.

Figure S5

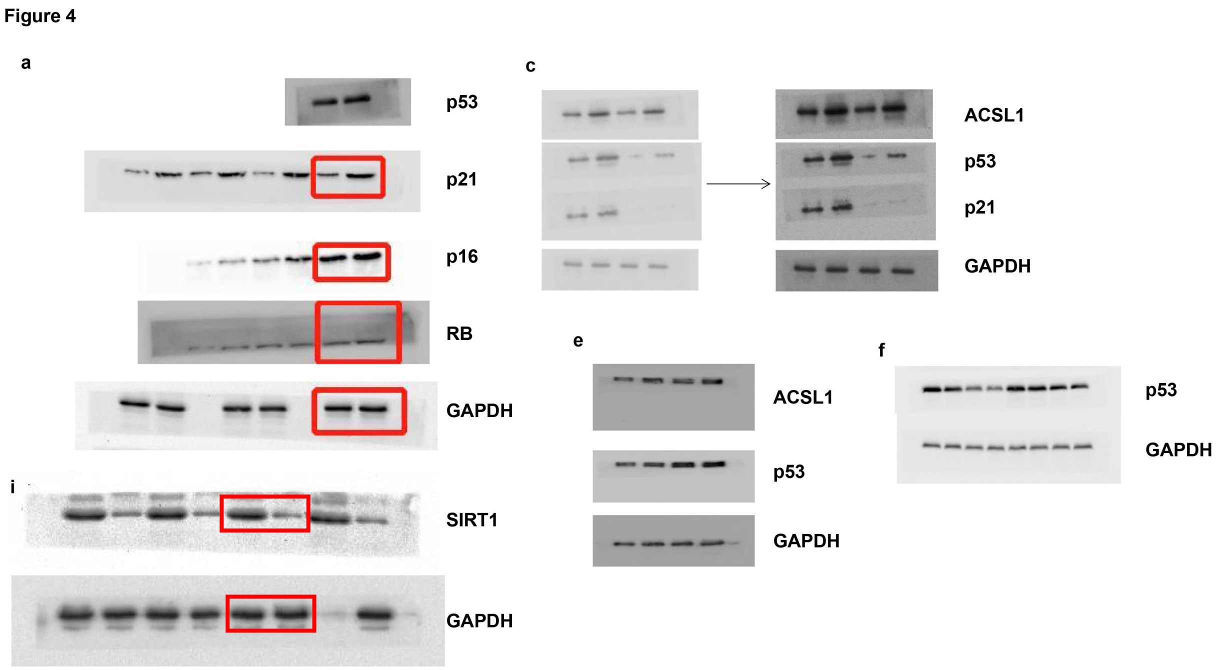

Supplementary Figure S5.

Gels and blots which are shown cropped in Figure 4a, c, e, f, i. Note: The blot was cut into multiple strips following the protein transfer. The edges of the PVDF membrane are visible. Here, bands containing the following regions were separately imaged: p53 (40-60 kDa), p21 (15-30 kDa), p16 (10-25 kDa), Rb (100-130 kDa), SIRT1 (100 -130 kDa), ACSL1 (60-90 kDa) and GAPDH (20-40 kDa).

Figure S6

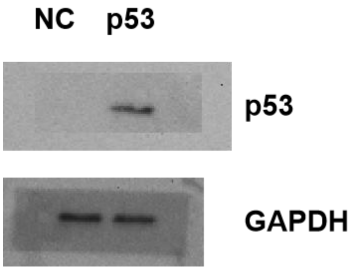

Supplementary Figure S6.

Gels and blots which are shown cropped in Figure S1. Note: The blot was cut into multiple strips following the protein transfer. The edges of the PVDF membrane are visible. Here, bands containing the 40-60 kDa and 20-40 kDa regions were imaged, respectively.
